# Oral manifestations as the first presenting sign of Crohn’s disease in a pediatric patient

**DOI:** 10.4317/jced.53914

**Published:** 2017-07-01

**Authors:** Ashley Eckel, Dale Lee, Gail Deutsch, Anthony Maxin, Dolphine Oda

**Affiliations:** 1 MD, PhD, University of Washington, Department of Laboratory Medicine, Seattle, WA; 2 MD, MSCE, Seattle Children’s Hospital, Division of Gastroenterology and Hepatology, Seattle, WA; 3 MD, Seattle Children’s Hospital, Department of Pathology, Seattle, WA; 4 Seattle Children’s Hospital, Seattle, WA; 5 BDS, MSc, University of Washington School of Dentistry, Department of Oral & Maxillofacial Surgery, Seattle, WA

## Abstract

Crohn’s disease (CD) is a chronic inflammatory disorder affecting the gastrointestinal (GI) tract. Although the GI tract is the primary site of involvement, many patients, particularly in pediatric cases, first present with non-intestinal manifestations, including oral lesions. Oral manifestations of CD in children occur in around 50-80% of cases, and about 30% of CD cases in children occur first in the mouth. Recognizing such oral lesions in the pediatric population, and requesting a biopsy, may expedite the diagnosis of CD. We describe a 15 year old male who presented with oral findings of multiple aphthous ulcers and plaques of pink papules of the buccal vestibule. We highlight the initial pathology findings, including non-caseating granulomas, sialadenitis, and a notable plasmacytosis, from biopsy of the left retromolar pad area, which triggered further testing for CD. We provide discussion of how CD was eventually diagnosed and treated and highlight the significance of the pathological findings in this case as they relate to the pathogenesis of CD.

** Key words:**Crohn’s disease, Inflammatory bowel disease, Oral manifestations, Pediatric, Granulomatous inflammation, Monotypic plasma cells.

## Introduction

Inflammatory Bowel Diseases (IBD) which include Crohn’s disease (CD) and ulcerative colitis (UC) are chronic and disabling diseases with a high and increasing incidence across continents (1). CD is an immune mediated chronic inflammatory disorder characterized by granulomatous inflammation that can occur from the oral cavity to the anus (2). Approximately 50-80% of pediatric patients with CD have oral changes including multiple superficial ulcers (aphthous ulcers) and cobblestone appearing pink papules on the buccal mucosa and vestibule (3). Of those, in more than 30%-60%, the oral findings may be the first manifestation of CD (3,4). In the pediatric population, failure to consider CD on the differential diagnosis of persistent multiple oral ulcers and other oral findings can delay necessary care for patients (3). We describe a 15 year old male who presented with multiple persistent oral ulcers and papules which were found to be the first manifestation of CD. We present this case with review of current literature pertaining to oral CD in pediatric patients. This case also offers an interesting pathology finding, highlighting the immunologic nature of the disease. Consistent with almost all cases of CD, non-caseating granulomas were found and were the trigger for further testing for CD; in this case, however, there was also a collection of monotypic plasma cells in the oral biopsy.

## Case Report

**Figure 1:**
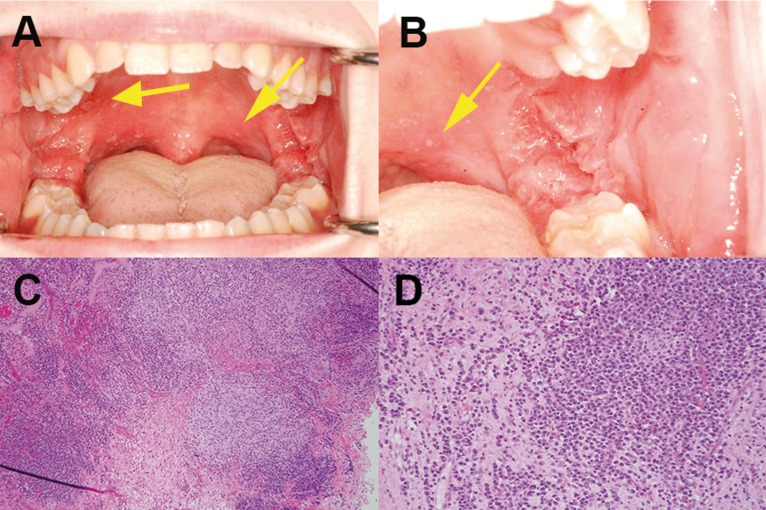
(A,B) Multiple small and superficial ulcers (yellow arrows) with white centers and red halos in the posterior soft palate of the patient’s oral cavity. Plaques of pink papules are present in the posterior buccal mucosa and bilateral retromolar areas. (C,D) Biopsy of the left retromolar pad area demonstrates mucosal ulceration with discrete non-caseating granulomas and plasmacytosis with patchy infiltrates of monotypic kappa light chain plasma cells.

A 15-year old Caucasian male initially presented to his family practice physician with palpable bilateral cervical lymph nodes. The enlargement of the nodes was interpreted to be of viral association and was treated conservatively. Approximately one month later, the patient developed episodic oral pain while eating which evolved into gingival bleeding, cheek swelling, and was associated with development of generalized superficial ulcers with clustering in the posterior soft palate and the vestibules. The patient also developed confluent flesh-colored papules in the bilateral posterior vestibule, buccal mucosa, and retromolar pad areas of his mouth (Fig. 1 A,B). He was referred to an Otolaryngologist who started the patient on a 10 day course of antibiotics and referred the patient to an oral surgeon for an incisional biopsy. Following results of the incisional biopsy of the left retromolar pad, the patient was next referred to Gastroenterology and underwent endoscopy and colonoscopy. Additional history obtained by the Gastroenterologist prior to endoscopic evaluation was significant for a ten pound weight loss over approximately one year associated with intermittent right-sided abdominal discomfort and episodes of blood on the toilet paper. The patient’s family history is notable for Crohn’s disease in a paternal grandmother, lymphocytic colitis in a paternal aunt, and ulcerative colitis in a paternal great-grandfather.

Fecal calprotectin prior to endoscopy was 477 mcg/g. Rheumatoid factor was normal. Erythrocyte sedimentation rate was 18 mm/hr. C-reactive protein was 13 mg/L. Serum protein electrophoresis demonstrated a broad gamma increase, with a non-specific pattern and no monoclonal proteins. Quantiferon gold test was negative for tuberculosis.

As requested by the oral surgeon, biopsy of the left retromolar pad area was performed.

Histologic sections demonstrated mucosal ulceration with discrete non-caseating granulomas, sialadenitis, and plasmacytosis with patchy infiltrates of monotypic kappa restricted light chain plasma cells (Fig. 1C,D). Testing for Epstein-Barr Virus was negative as well as fungal, mycobacterial, and spirochete special stains. A diagnosis of non-caseating granulomatous inflammation was rendered with a comment mentioning Crohn’s disease as a possible etiology.

**Figure 2:**
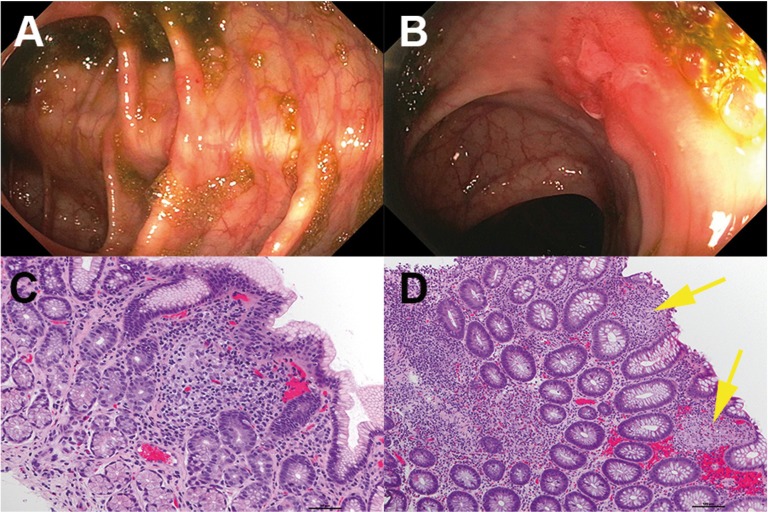
(A,B) Areas of mildly erythematous mucosa and multiple aphthous ulcers with scattered areas of white exudates and mucosal hemorrhages in the colon, similar to those noted in the oral cavity. (C) Biopsy of gastric mucosa with non-caseating granuloma. (D) Right colon with diffuse acute and chronic inflammation, reactive injury, and multiple non-discrete granulomas (yellow arrows).

On subsequent examination by endoscopy, the upper gastrointestinal tract was notable for mild patchy erythema in the duodenum. The entire colon demonstrated scattered areas of mildly erythematous mucosa and multiple aphthous ulcers similar to those noted in the oral cavity. Multiple biopsies were taken. The terminal ileum contained a patchy area of mucosa that was mildly erythematous. Scattered white exudates and mucosal hemorrhages were appreciated and, again, multiple biopsies were taken. The perianal and digital rectal examinations were normal (Fig. 2 A,B). MRI enterography demonstrated no radiographic evidence of bowel inflammation.

Biopsies taken at the time of endoscopy were remarkable for mild to moderate chronic active inflammation with multifocal discrete non-caseating granulomas in both the upper and lower gastrointestinal tract similar to those of the oral cavity lesions. The stomach and colon had multifocal discrete granulomas (Fig. 2C,D). The esophagus and duodenum were more mildly affected. The terminal ileum had focal prominent surface infiltration by neutrophils with underlying granulation changes and subtotal villous architecture atrophy.

The differential diagnosis for this patient’s clinical presentation included CD, infectious diseases (fungal, mycobacterial, spirochetes), drug-induced reactions, response to foreign body, nutritional deficiencies, and rheumatological disease. A variety of infectious or drug-related conditions have been shown to clinically mimic CD, although those etiologies often present more acutely. Granulomas can occur in infectious diseases including tuberculosis and Yersinia pseudotuberculosis.

Based on results of the oral biopsy, endoscopic evaluation and associated biopsies, and laboratory values, a diagnosis of Crohn’s disease was rendered. After discussion with the patient and family, the decision was made to start both corticosteroids and enteral nutritional therapy, with 80% of daily calories from an intact protein formula (5). The patient and family were interested in considering nutritional therapy long-term. The patient began a steroid wean and has not had recurrence of oral lesions or gastrointestinal symptoms. The decision to start maintenance immunosuppressive therapy was made six weeks after diagnosis as the family decided that primary nutritional therapy would be difficult to maintain long-term.

## Discussion

Crohn’s disease is a chronic inflammatory bowel disease believed to involve interplay of genetic, immunologic, and environmental factors. It commonly begins gradually, but may also start abruptly. Two age peaks in occurrence are described: one among early adulthood and the other in patients over 60 years of age (6,7). Children and adolescents with CD often have more extensive disease with a more severe disease course than adults. Although the GI tract is the primary site of involvement in CD, many cases, particularly in pediatric patients, first present with non-intestinal manifestations, including oral lesions. Younger pediatric patients often also present with weight loss, delayed growth, or failure to thrive. As with the patient presented here, younger age at diagnosis (younger than 20 years) is associated with greater prevalence of a family history of CD (8).

Studies have shown that oral manifestations of CD in children occur in around 50%-80% of cases, and about 30% of CD cases in children occur first in the mouth (3). One study suggested as high as 60% of cases of pediatric CD have oral symptoms as the first presenting sign of the disease (9). Oral lesions can precede, occur concurrently, or follow the onset of abdominal symptoms, although synchronous observation is most commonly described. Failure to include IBD, particularly CD, on the differential for oral manifestations can lead to delay in diagnosis and treatment for patients or extensive unnecessary workups.

The most common sites for clinical presentation of oral lesions in CD are the lips, gingiva, vestibular sulci and buccal mucosa (9). Mucogingivitis occurs in about 25% of cases, followed by multiple and persistent superficial oral ulcers simulating minor apht-hous ulcers, which occur in about 8% of the cases (10). Cobblestone papules of the buccal mucosa and vestibule occur in about 6% of the patients (10). These findings may be associated with pain, impairment of oral function, and psychosocial stress (9). Other non-specific oral findings of CD include angular cheilitis, persistent submandibular lymphadenopathy, gingivitis, and pe-riodontal disease (4,11).

The first step in treating oral lesions is to assess for, and control, intestinal disease (4). It is also important to form a complete differential diagnosis for the oral manifestations, including side effects of drugs, nutritional deficiencies, infections and other granulomatous diseases with oral involvement (4). If the oral findings are asymptomatic, no particular treatment for the oral lesions is necessary and they will resolve over time in association with the treatment of gastrointestinal disease. The diagnosis of CD is ultimately made by a combination of clinical, laboratory, radiographic, and endoscopic observations. The clinical course of CD is characterized by exacerbation and spontaneous, medication, or dietary therapy induced remission. General treatments include sulfa-type drugs, i.e. sulfasalazine; dietary therapy, or immunosuppressive agents such as systemic steroids, azathioprine, methotrexate, or anti-TNF-alpha antibodies.

While the exact causes of Crohn’s disease remain unknown, some studies have postulated that changes in the immune system and exposure to environmental risk factors, including responses to gastrointestinal bacteria, may be triggers of CD (12). Dysregulation of various components of the immune system can be seen in the gut of patients with CD. This dysregulation is thought to be sustained by increased local proinflammatory cytokine products and by defects in counter-regulatory mechanisms (13).

In this patient’s case, the histological suggestion of CD was largely based on the finding of the non-caseating granulomas. The occurrence of focal inflammation simultaneous with severe patchy inflammation in the set of biopsies also gave a significant discriminative value in favor of CD. In a study of patients with oral CD, non-caseating granulomas were found in 100% of the oral biopsy specimens taken from the oral lesions, highlighting the value of the easily accessible oral mucosa as a potential site for harvesting diagnostic material, especially in pediatric patients (10).

This case also presented a notable finding of predominantly monotypic, kappa restricted, plasma cells infiltrating the biopsy tissue. While in CD the gut is known to be massively infiltrated with B cells and plasma cells, the plasma cells are usually polyclonal. The role of the plasma cells in the pathogenesis of gut tissue damage is largely unknown. In CD, plasma cells have been shown to produce non-organ-specific antibodies, which might contribute to complement deposition and destruction of gastrointestinal epithelial cells (14,15). It is always a concern when plasma cells appear to be monotypic, giving the impression of a neoplastic cell proliferation such as a plasma cell neoplasm or lymphoid neoplasm with plasmacytic differentiation; however, the age of the patient made these diseases highly unlikely. Additionally, malignant lymphomas with plasmablastic differentiation of the mouth might also be included on the differential, but are more commonly described in immune compromised patients.

In conclusion, we describe a 15 year old male with persistent multiple oral aphthous ulcers and plaques of pink papules of the buccal vestibule that were the first manifestation of CD. Recognizing such oral lesions in the pediatric population and requesting a biopsy of the accessible papules and/or the superficial ulcers may help expedite the diagnosis of CD.
